# Evaluation of the efficacy of mineral trioxide aggregate and bioceramic putty in primary molar pulpotomy with symptoms of irreversible pulpitis (a randomized‐controlled trial)

**DOI:** 10.1002/cre2.700

**Published:** 2022-12-04

**Authors:** Ibrahim Alnassar, Mohamed Altinawi, Mohammad Salem Rekab, Hasan Alzoubi, Anas Abdo

**Affiliations:** ^1^ Department of Pediatric Dentistry, College of Dentistry Damascus University Damascus Syria; ^2^ Department of Restorative Dentistry and Endodontics, College of Dentistry Damascus University Damascus Syria

**Keywords:** Bioceramic, irreversible pulpitis, MTA, pulpotomy

## Abstract

**Objectives:**

Preserving the primary teeth is important, as they play an important role in the integrity of the dental arch, the development of the craniofacial complex, speech, and chewing. This study aimed to evaluate the effectiveness of both Mineral Trioxide Aggregate (MTA) and Bioceramic putty in primary molar pulpotomy with symptoms of irreversible pulpitis.

**Materials and Methods:**

In this study, 40 s primary mandibular molars in 40 healthy children aged 6−8 years were examined and classified into 2 groups according to the material: group A, with 20 primary molars capped by MTA, and group B, with 20 teeth capped by Bioceramic putty. Clinical and radiographic evaluation of the treatment results was carried out after 1 week, 3 months, 6 months, 9 months, and 1 year.

**Results:**

Clinical and radiological success rates in the MTA group reached 95%, where a case of failure was observed after a year of follow‐up. In the Bioceramic group, the success rate reached 100% after a year of follow‐up, without any statistically significant differences between groups (*p* = .311).

**Conclusions:**

Pulpotomy using biocompatibility materials (MTA‐Bioceramic) in primary molars with symptoms of irreversible pulpitis is considered effective due to the better advantages of the use of Bioceramic over MTA. This clinical trial was approved by Australian New Zealand Clinical Trials (12621001631897).

## 1 INTRODUCTION

The main objective of pulp treatment is to maintain the health and integrity of the oral tissues. Early loss of primary teeth leads to malocclusion and esthetic, phonetic, and functional problems (Evangelista et al., 2022).

Root canal treatment is indicated in primary teeth that show symptoms of irreversible pulpitis, but it is a challenge, time‐consuming, and expensive, in addition to the fact that it requires definitely positive cooperation from the children, as their behavior can affect the treatment results positively or negatively. (Alzoubi et al., 2021; Fuks et al., 2019).

The dissection of the molar root canals increases complications, which may lead to tooth extraction. Excellent skills are required to perform endodontic treatment, taking into account possible damage to the permanent tooth bud during treatment due to the files used or filling materials (Pinheiro et al., 2012).

Vital pulp therapy using biocompatibility materials is an option as an alternative to pulpectomy in cases of irreversible pulpitis, where studies have shown a weak correlation between the histological status of the pulp and the symptoms that the patient complains about (Ricucci et al., 2019).

Use of biocompatibility materials has led to a high success rate in cases of irreversible pulpitis, such as mineral trioxides, as it was applied in many studies on permanent molars of patients with irreversible pulpitis (Koli et al., 2021; Qudeimat et al., 2017).

Mineral Trioxide Aggregate (MTA) and Bioceramic were presented as alternative biocompatibility materials in primary molar pulpotomy, as use of these materials showed a high success rate (Hegde & Naik, 2005; Lei et al., 2019).

In 2007, a group of Canadian researchers introduced a ready‐to‐use, no‐mix Bioceramic based on calcium silicate. This material is available in three forms: Bioceramic Root Repair Material Putty (BC RRM Putty, fast set putty), BC RRM Past (Bioceramic Root Repair material, a syringable paste), and BC Sealer (Bioceramic sealer) (Xavier et al., 2019).

Bioceramic consists of Tricalcium silicate, Dicalcium silicate, zirconium oxide, tantalum pentoxide, and Calcium phosphate monobasic as well as filler and thickening agents (Debelian & Trope, 2016).

The histological pulp structure of primary teeth is similar to that of permanent ones (Fuks et al., 2016), where many studies revealed the occurrence of the same vascular/immune responses in both primary and permanent teeth when their pulp tissue was exposed to bacterial invasion because of caries (Rodd & Boissonade, 2005, 2006).

Several studies have evaluated the efficacy of biocompatibility materials in primary molar pulpotomy with irreversible pulpitis (Asgary et al., 2021; Memarpour et al., 2016), but no study has evaluated the efficacy of both MTA and BC putty in such cases.

Therefore, the main aim of this study was to evaluate the success rate of pulpotomies on primary molars with irreversible pulpitis and the secondary aim was to compare the effectiveness of MTA and BC putty.

## 2 MATERIALS AND METHODS

### 2.1 Sample size and power calculation

The sample size was determined using a sample size calculation program (PS Power and Sample Size Calculation Program, Version 3.0.43). Sample size calculation produced a required sample size of 20 primary molars per group to detect a significant difference (90% power, two‐sided 5% significance level).

### 2.2 Study population and inclusion criteria

A total of 40 s primary mandibular molars in 40 patients were assessed for the study and the patients were invited to participate in the study on fulfillment of the following inclusion criteria:

1. Children aged 6−8 years with physiological absorption of less than one‐third of the root.
2. Presence of extended carious lesion with symptoms of irreversible pulpitis.
3. Absence of clinical symptoms or signs of pulp necrosis (fistula, movement of the tooth, and swelling). No more than one infected molar in the same quarter of the jaw.
4. Absence of radiographic signs of pulp necrosis (pathological internal or external absorption, periapical lesion).
5. Definitely positive or positive children according to the Frankle scale and restorable teeth.

The exclusion criteria were as follows:

1. Children with systematic or mental disorders.
2. Presence of any clinical and radiographic signs that indicate pulp necrosis, such as internal or external root resorption, inter‐radicular and/or periapical bone destruction, the existence of periapical translucence, swelling, or sinus tract, and tenderness to percussion.
3. Teeth that are not to be isolated by the rubber dam and children who have poor oral hygiene.

### 2.3 Randomization

**Figure 1:**
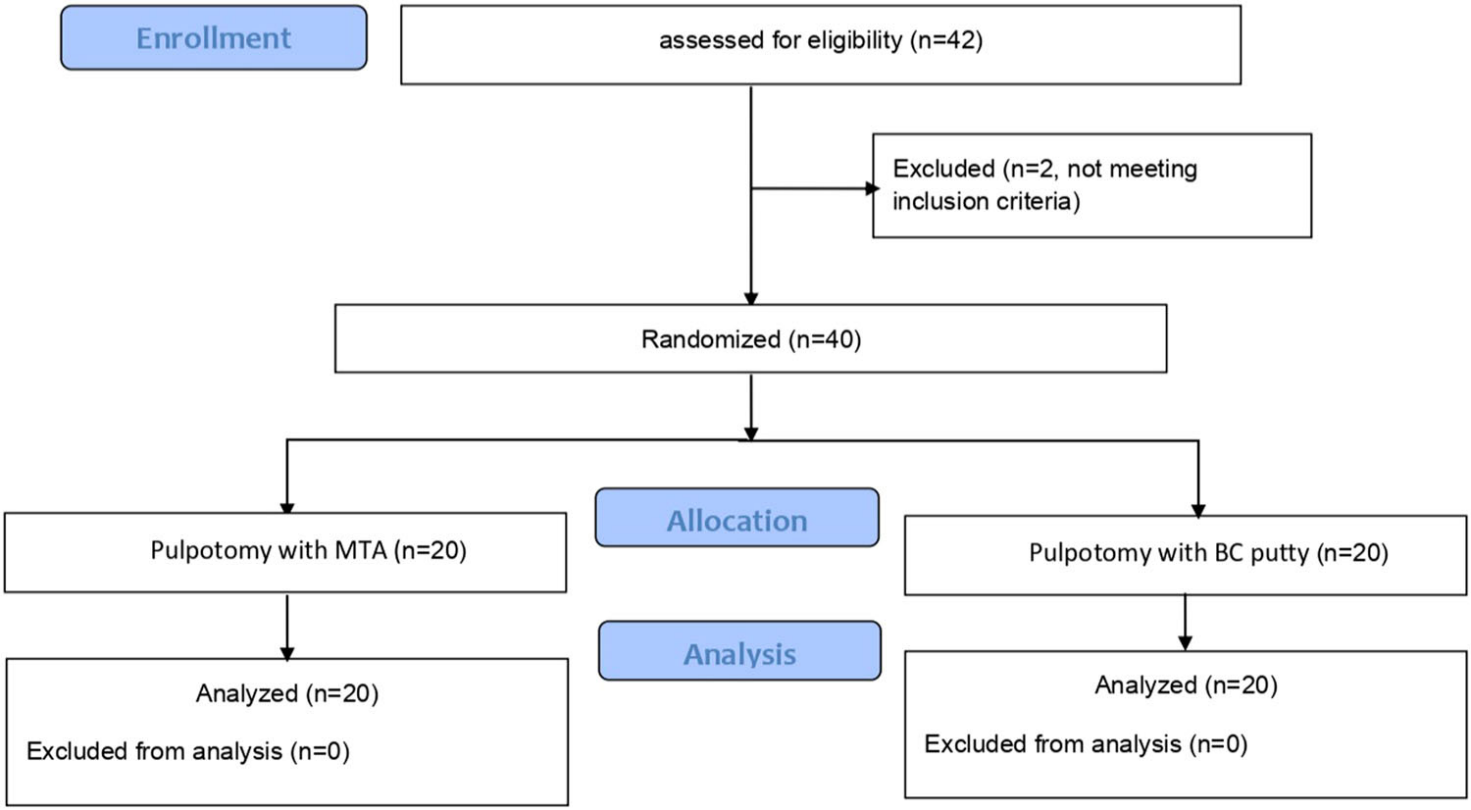
CONSORT flowchart

This randomized clinical trial has been developed according to CONSORT statement guidelines (Figure 1). The studied sample was randomly distributed according to http://www.randomization.com into two groups:

Group (A) represents the control group, which was treated with MTA, and Group (B) represents the experimental group, which was treated with BC putty.

A double‐blinded design was also adopted in this study so that both the patient and the examiner were unaware of the applied material.

### 2.4 Treatment procedure

All dental treatments were provided at the Damascus‐Faculty of Dentistry‐Department of pediatric dentistry. After applying the local anesthetic gel, lidocaine was administered 2% with 1:80000 epinephrine, and isolation was done with a rubber dam.

Enamel and dentin were removed using a high‐speed diamond bur with a water‐cooled handpiece, ensuring the removal of the caries area near the pulp by using an excavator from the periphery toward the center to reduce the amount of bacterial contamination.

The pulp chamber access was refined with Endo‐z (Dentsply Maillefer, Ballaigues, Switzerland) with water cooling and then the remainder of the coronal pulp was removed using a sharp excavator.

Hemostasis was achieved with cotton pellets moistened with 2.5% sodium hypochlorite for 2 min, and the process was repeated, if necessary, until the bleeding stopped within 10 min. If hemostasis was not achieved, the patient was excluded and pulpectomy treatment was performed.

Then, BC putty (Well‐Root pt) or MTA angelus (MTA angelus) was applied depending on the group to which the patient was assigned. A base layer of glass ionomer cement was applied (Fuji IX®, GC Corporation, Tokyo, Japan) and then the tooth was restored with a stainless‐steel crown (Kids Crown; Shinhung, Seoul, Korea).

### 2.5 Outcome assessment

**Figure 2:**
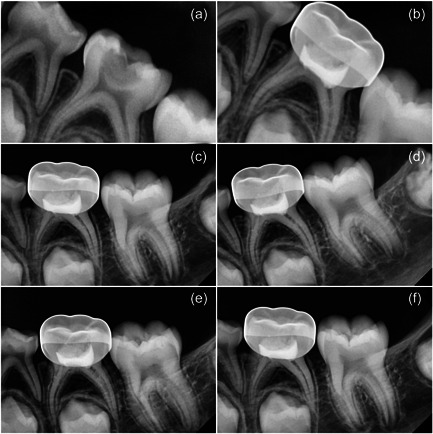
Bc putty follow‐up (a) Diagnostic image, (b) 1‐week follow‐up, (c) 3‐month follow‐up, (d) 6‐month follow‐up, (e) 9‐month follow‐up, and (f) 12‐month follow‐up

**Figure 3:**
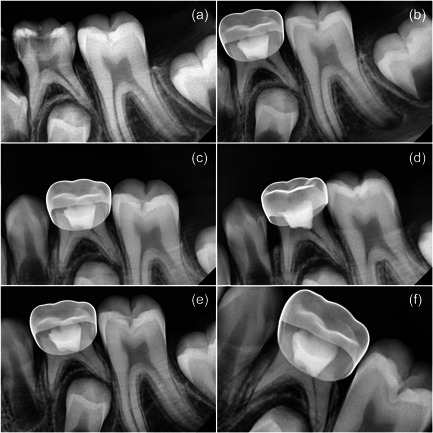
MTA follow‐up (a) Diagnostic image, (b) 1‐week follow‐up, (c) 3‐month follow‐up, (d) 6‐month follow‐up, (e) 9‐month follow‐up, and (f) 12‐month follow‐up. MTA, Mineral Trioxide Aggregate.

Clinical and radiographic assessment was carried out after 1 week and at 3, 6, 9, and 12 months (Figures 2 and 3) by two specialists who were unaware of the applied material.

The treatment was considered clinically successful in the absence of pain, swelling, fistula, and pain on percussion and bites.

Treatment was considered successful radiologically in the absence of periodontal ligament widening, and internal or external root resorption, in addition to evaluating the presence of radiolucency in the furcation area according to the following scores:

Score 0: no radiolucency; score 1: radiolucency between ¼ of furcation to periapical areas; score 2: radiolucency between ¼ and ½ of furcation to periapical areas; and score 3: radiolucency more than ½ of furcation to periapical areas. Where the treated teeth with a score of 1 or 2 considered successfull according to the previous criteria.

Statistical analysis was performed using SPSS 21.0 software (IBM, Armonk, NY, USA). The data were analyzed using the *χ* ^2^ Test. The significance level was set at 5% for the present study (*p* < .05).

## 3 RESULTS

The study sample consisted of 40 cases of pulpotomy performed for 40 s primary mandibular molars in 40 children of both sexes; their ages ranged between 6 and 8 years, with an average age of 6.93 ± 0.75 years in the MTA group and 7.81 ± 0.41 years in the BC putty group, as shown in Table 1. The *χ* ^2^ Test was applied to compare the two groups.

**Table 1:** Basic sample characteristics

Group	Sex	*N* (%)	Age: mean ± SD
MTA	Male	9 (45%)	6.93 ± 0.75
	Female	11 (55%)	
Bioceramic	Male	10 (50%)	7.18 ± 0.41
	Female	10 (50%)	

One case of clinical and radiological failure was recorded, where the appearance of pain on the bite was observed, in addition to large translucency in the furcation area in the MTA group at the 3‐month follow‐up period (Table 2).

**Table 2:** Comparisons of success and failure rates during follow‐up periods

Follow‐up periods	Assessment	Group	Success	Failure	*p* Value
1 week	Clinical	MTA	100%	0%	‐
		Bioceramic	100%	0%	
	Radiographic	MTA	100%	0%	‐
		Bioceramic	100%	0%	
3 months	Clinical	MTA	95%	5%	0.311
		Bioceramic	100%	0%	
	Radiographic	MTA	95%	5%	0.311
		Bioceramic	100%	0%	
6 months	Clinical	MTA	95%	5%	0.311
		Bioceramic	100%	0%	
	Radiographic	MTA	95%	5%	0.311
		Bioceramic	100%	0%	
9 months	Clinical	MTA	95%	5%	0.311
		Bioceramic	100%	0%	
	Radiographic	MTA	95%	5%	0.311
		Bioceramic	100%	0%	
12 months	Clinical	MTA	95%	5%	0.311
		Bioceramic	100%	0%	
	Radiographic	MTA	95%	5%	0.311
		Bioceramic	100%	0%	

Abbreviation: MTA, Mineral Trioxide Aggregate.

During the 12‐month follow‐up period, the success rate was 95% in the MTA group, while it was 100% in the BC putty group. The results of the chi‐square test showed that there was no statistically significant difference between the two study groups (*p* = .311) as shown in Table 2.

## 4 DISCUSSION

Irreversible pulpitis of primary teeth are usually treated with pulpectomy its inflamed and healthy pulp, as this procedure is considered invasive, nonbiological, and results in a loss in the repair and regenerative properties that characterize the vital pulp therapy (Wolters et al., 2017).

The pulpectomy procedurerequires more time and effort in children and the experience of the dentist compared to pulpotomy treatment, these reasons may lead to a decision to extract the primary tooth, which leads to a loss in the function of the tooth (McDOUGAL et al., 2004; Smaïl‐Faugeron et al., 2018).

Therefore, vital pulp treatment techniques have been proposed to treat cases of irreversible pulpitis where this technique is characterized by preserving the immune and vital functions of the pulp, as well as this procedure is simple and avoids complications resulting from the complex anatomy of the root canal anatomy, and less pain‐related treatment compared to root canal treatment (Simon et al., 2013; Wolters et al., 2017).

The effectiveness of vital pulpotomy has been proven in the treatment of teeth affected by symptoms and signs of irreversible pulpitis, whether in permanent or primary teeth. Taha et al found a 100% success rate after a year of follow‐up and a 92% success rate after three years, where pulpotomy was performed by MTA in permanent molars affected by irreversible pulpitis (Taha & Khazali, 2017).

Cushley et al also evaluated, in a systematic review, the success of pulpotomy in teeth affected by irreversible pulpitis, where they found a clinical success rate of 97.4% and a radiological success rate of 95.4% after a follow‐up period of 12 months (Cushley et al., 2019).

Memarpour et al. (2016) evaluated the effectiveness of a calcium‐enriched mixture (CEM) in primary molar pulpotomy with irreversible pulpitis, where they found a success rate of 92.8% after a year of follow‐up. These results are in agreement with the results of pulpectomy, for which success rates ranging from 70% to 90% have been reported (Barcelos et al., 2012).

The study sample consisted of 40 s primary mandibular molars to avoid differences resulting from the type of teeth, and molars with irreversible pulpitis (based on the symptoms reported by the patient of spontaneous and continuous pain that requires an analgesic to be given to the child) were included.

The time required for hemostasis to occur was between 5 and 10 min after removing the coronal pulp; the case in which the time exceeded 10 min was excluded and shifted to a complete pulpectomy, wherein bleeding that exceeded 10 min was considered to be indicative of severe pulpitis that requires pulpectomy, depending on the classification of World Wolters (Wolters et al., 2017).

Sodium hypochlorite was used as a disinfection solution with a concentration of 2.5% after removing the coronal pulp, based on the recommendations of the American Association of Endodontists, according to which sodium hypochlorite solution with a concentration ranging from 0.5% to 5.25% should be used when treating vital pulp (Bogen et al., 2008).

Disinfection with sodium hypochlorite is considered safe without causing damage or toxicity to pulp cells or their proliferation and differentiation and it did not show any negative effect on dentin bridge formation; a concentration of 2.5% was chosen as the median value in this study (Duncan et al., 2021).

In this study, MTA was used, which is considered the gold standard in the context of vital pulp therapy due to its high biocompatibility and high success rate (Hussain & Bashar, 2022).

Stainless‐steel crowns were used for final restoration due to their high sealing capacity and durability. The quality of the final restoration plays an important role in improving the treatment prognosis (Demarco et al., 2005).

Some studies have shown that coronal sealing is possibly to be more important than the biocompatibility materials used in the context of vital pulp therapy (Qudeimat et al., 2017), and the high success rate in this study may be attributed to the excellent quality of the coronal seal.

The success rate of Bioceramic putty, clinically and radiologically after a 1‐year follow‐up, was 100%, while the success rate of MTA was 95%. The clinical success depended on the absence of spontaneous pain on percussion and the absence of a fistula.

The radiographic success depended on the absence of internal or external absorption and the absence of periodontal ligament widening, in addition to an assessment of radiolucency in the root furcation area, where this area was adopted in the evaluation due to the presence of lateral canals in the junction area (Smaïl‐Faugeron et al., 2018).

No study has compared MTA with Bioceramic putty in primary molar pulpotomy diagnosed with irreversible pulpitis, but the results of this study are in agreement with the results of the Memarpour et al study, with a difference in the material used in a pulpotomy; they used CEM, and reported a success rate of 92% after a year of follow‐up (Memarpour et al., 2016).

Asgary et al also evaluated CEM in primary molar pulpotomy with irreversible pulpitis; a success rate of 100% was found after a 34‐month observation period, and they also achieved a success rate of 100% in permanent molar pulpotomy (Asgary et al., 2021).

## 5 CONCLUSION

Both MTA and BC putty showed promising results in the context of primary molar pulpotomy with symptoms of irreversible pulpitis, and this procedure can be considered as an option in the treatment of these cases as a conservative choice.

## AUTHOR CONTRIBUTIONS

Ibrahim Alnassar conceived the idea and provided the treatment. Hasan Alzoubi and Anas Abdo contributed to the writing and documenting. Mohamed Altinawi and Mohammad Salem Rekab conceived the idea and supervised the treatment.

## CONFLICT OF INTEREST

The authors declare no conflict of interest.

### ETHICS STATEMENT

The study protocol was approved by the Scientific research and Postgraduate Board of Damascus University Ethics Committee, Damascus, Syria (IRB No. UDDS‐4030‐26082019/SRC‐1450). The study protocol was also enrolled at the Australian New Zealand Trials (12621001631897). A detailed information sheet in simple nontechnical language was provided in advance, and parents/guardians were requested to sign an informed consent. The patients and parents were blinded to the study as they were not provided any information about the treatment group to which they were assigned.

## Data Availability

The data that support the findings of this study are available on request from the corresponding author. Data are available upon request due to privacy from the corresponding author.
